# Librarian integration into health care conferences: a case report

**DOI:** 10.5195/jmla.2020.803

**Published:** 2020-04-01

**Authors:** Carrie Price, Sapna R. Kudchadkar, Pragyashree Sharma Basyal, Archana Nelliot, Madison Smith, Michael Friedman, Dale M. Needham

**Affiliations:** Librarian, Welch Medical Library, Johns Hopkins University School of Medicine, Baltimore, MD, cprice17@jhmi.edu, https://orcid.org/0000-0003-4345-3547; Physician, Department of Anesthesiology and Critical Care Medicine, Department of Physical Medicine and Rehabilitation, and Department of Pediatrics, Johns Hopkins University School of Medicine, Baltimore, MD, sapna@jhmi.edu; Staff, Division of Pulmonary and Critical Care Medicine, and Division of Geriatric Medicine and Gerontology, Johns Hopkins University School of Medicine, Baltimore, MD, prag.sharma@jhmi.edu, http://orcid.org/0000-0002-7180-6955; Resident, Department of Pediatrics, Penn State Hershey Medical Center, Hershey, PA, anelliot@pennstatehealth.psu.edu; Staff, Division of Pulmonary and Critical Care Medicine, Johns Hopkins University School of Medicine, Baltimore, MD, msmit354@jhmi.edu; Physical Therapist, Department of Physical Medicine and Rehabilitation, Johns Hopkins Hospital, Baltimore, MD, mfried26@jhmi.edu; Professor, Division of Pulmonary and Critical Care Medicine, Department of Physical Medicine and Rehabilitation, Johns Hopkins University School of Medicine, Baltimore, MD; and School of Nursing, Johns Hopkins University, Baltimore, MD, dale.needham@jhmi.edu

## Abstract

**Background:**

Health care continuing education conferences are important educational events that present opportunities for structured learning, interactive sharing, and professional networking. Conference presenters frequently cite published literature, such as clinical trials, to supply an evidence-based foundation, with presenters’ slides often shared with conference attendees. By using social media, these conferences can have greater impact, assist in supporting evidence-based clinical practice, and increase stakeholder engagement.

**Case Presentation:**

The authors present a case of embedding a health sciences librarian into the Annual Johns Hopkins Critical Care Rehabilitation Conference. The librarian served multiple roles, including social media ambassador, conference exhibitor, and presenter. We explore how these roles contributed to the field of early rehabilitation research through information dissemination and education. We also address best practices for librarian support of the conference, with a discussion of tools, platforms, and work flows that were beneficial.

**Conclusions:**

Librarian integration facilitated education about bibliographic literature database content, database searching, critical appraisal, and reporting of search methodology. Additionally, the librarian contributed to real-time distribution of scholarly literature through proficiency with web platforms, citation management programs, and social media. Librarians’ expertise in information organization and dissemination, as well as various technology platforms, make them a valuable addition to health care conferences.

## BACKGROUND

Health sciences librarians strive to build enduring partnerships with their stakeholders. Boundary spanning [1], the formulation of multidisciplinary or multi-organizational collaborations, is common in the health sciences library profession [2]. Working among various clinical teams can be “fascinating and transformative,” and earning a reputation as a “great partner” paves the way for meaningful future collaboration [2]. Librarians are naturally inclined helpers due to the nature of their profession. “Enthusiastic and confident librarians are a key element in reinventing the clinical librarian role in today’s highly information-based practice of medicine” [3], which remains true especially as librarians discover novel ways in which to offer services and collaborate with their users.

In this case report, the authors describe one librarian’s experience as an embedded information professional in an annual health care continuing education conference whose goal is to promote information literacy and disseminate the conference’s collective cited evidence through a social media platform and a conference bibliography.

The Annual Johns Hopkins Critical Care Rehabilitation Conference brings together health care professionals who are interested in evidence-based education and implementation of early rehabilitation for critically ill patients [4]. The first conference was held in 2012 and drew approximately 300 attendees. The conference aims to “bridge the interdisciplinary gap from research to the bedside, and bring together clinical experts,…creating a culture based on proactive rehabilitation” [5] for better patient outcomes. Entering its 8th year in 2019, the conference has grown rapidly, more than doubling its attendance in 2018 to over 700 attendees from across the United States and around the globe. Its thematic tracks have expanded to include a dedicated pediatric track, chaired by a pediatric critical care physician, in addition to the original adult track, chaired by an adult critical care physician. The conference, held on the Johns Hopkins Medical Campus, is multidisciplinary and includes physical therapists, occupational therapists, speech language pathologists, nurses, respiratory therapists, physicians, and psychologists. While the first conference was 2 days long, the scope and coverage of additional topics eventually necessitated 3 days. The conference includes patient and family interviews, vendors and exhibitors, poster and oral presentations, and a social and networking event.

In 2015, an academic health sciences librarian attended the fourth annual conference. The librarian was the liaison for two of the conference’s sponsoring groups: the Division of Pulmonary and Critical Care Medicine and the Department of Physical Medicine and Rehabilitation at Johns Hopkins University and Medical Institutions, along with Johns Hopkins Hospital Nursing. The initial motivation for librarian attendance was to learn more about these departments and their information needs, research interests, and literature landscape.

The 2015 conference gave rise to significant professional networking and engagement, both on a social media platform and in person. Nurses and rehabilitation clinicians readily engaged with physicians and psychologists. Attendees were excited to learn how they could be a catalyst for implementation of early rehabilitation at their home institutions. Envisioning the prospect of a collaborative role, the librarian inquired with the conference activity director to request formal inclusion in the 2016 conference. The original objective for librarian integration was to share presenters’ cited references from their slides in real-time, including hyperlinks in tweets, to promote knowledge of scholarly literature. In 2017, the conference organizers invited the librarian to become a conference exhibitor, along with other professional and commercial exhibitors, with the aim of promoting information literacy through handouts and in-person discussions.

## CASE PRESENTATION

### “Live Tweeting the Meeting”: librarian as social media ambassador

In recent years, Twitter has gained prominence in health care and become a commonly used platform for “live tweeting the meeting” [6], making “information from the meeting accessible to a global audience” [7], despite barriers to attending in person, such as cost, travel, and time constraints. In 2018, Twitter had an estimated 126 million active users on a daily basis and 330 million active users monthly [8, 9]. Twitter can be accessed through the web or a smartphone app and uses hashtags to improve findability and impact of content [10]. Twitter allows users to reply, like, and retweet content; write text; and embed photos, short videos, polls, and hyperlinks.

Early in the conference’s history, the organizers incorporated Twitter to help with dissemination of and wider engagement with conference content. According to a survey by Mohammadi et al., higher education professionals use Twitter to obtain information, share information, expand professional networks, contribute to a wider conversation, promote their organizations, communicate about academic events, communicate the results of their research, and engage in educational purposes [11]. The organizers also recognized Twitter would enhance discoverability by incorporating a hashtag for tweets related to the content of the conference. The initial goal for implementing social media into the conference was to circulate evidence for best practices while simultaneously creating a lasting community of clinicians with an interest in early rehabilitation of the critically ill.

In the conference’s earliest years, one designated social media ambassador used Twitter to share information about the schedule, presenters’ Twitter handles, and hyperlinks to scholarly literature. All conference tweets were sent from the Twitter account @ICURehab and included the hashtag #ICURehab. Conference attendees and others who engaged via Twitter were also encouraged to use the hashtag #ICURehab so that their tweets would be more easily discoverable.

Due to the conference’s growth, additional social media ambassadors were appointed in later years. The librarian was added as a second member of the Social Media Ambassador Team starting in 2016. The librarian’s primary role was to effectively disseminate links to published literature through Twitter, allowing the other social media ambassador to focus on tweeting photos of presenters and their slides and providing practice-related commentary. In 2017 and 2018, the pediatric critical care physician chaired the Social Media Ambassador Team, which now included three additional members, each attending different tracks or workshops to increase Twitter coverage. The social media chair also gave a Twitter primer presentation at the start of the conference so that attendees could learn how to download the Twitter app and start tweeting. Those attendees who chose to engage on Twitter could reply to conference tweets, connect with presenters, or retweet relevant content to their followers.

Conference presenters provided references as part of their slide presentations and were given a deadline to submit slides approximately one month prior to the conference. During the years of librarian involvement (2016–2018), conference organizers shared these slides with the librarian through a cloud storage program. The librarian aggregated the slides’ cited references into Paperpile [12], a citation management program that is a Google Chrome [13] browser plugin. Paperpile integrated easily with the Chrome browser so that references could be added to the bibliography from PubMed, Google Scholar [14], or the web with the click of a button. Most references were compiled prior to the conference and then exported into a bibliography, including a digital object identifier (DOI) [15] or PubMed or PubMed Central [16] hyperlink where possible. The bibliography was then shared with the Social Media Ambassador Team through Google Drive [17] and email just prior to the conference.

The Social Media Ambassador Team was advised to keep the bibliography open on their laptops during the conference so that whenever a presenter mentioned an article, it could be easily identified and shared through Twitter, along with any relevant commentary. In the instance that a presenter’s reference was not part of the bibliography, the team used several platforms for discovery to add or tweet them, including PubMed and Google Scholar. The Social Media Ambassador Team used PubMed, PubMed Central, open access journal, or DOI hyperlinks in their tweets when possible, since these interfaces have the highest likelihood of being accessible to the public without a paywall.

Shortly after completion of the conference, the librarian reviewed any additional content and the final Twitter transcript. Subsequently, the librarian presented the conference organizers with a complete bibliography of references for posting on the conference website [5] and sharing with attendees and other interested parties via email. For the 2018 conference, this bibliography contained more than 750 references, a number that continues to increase each year with the growth of the conference content.

**Figure 1:**
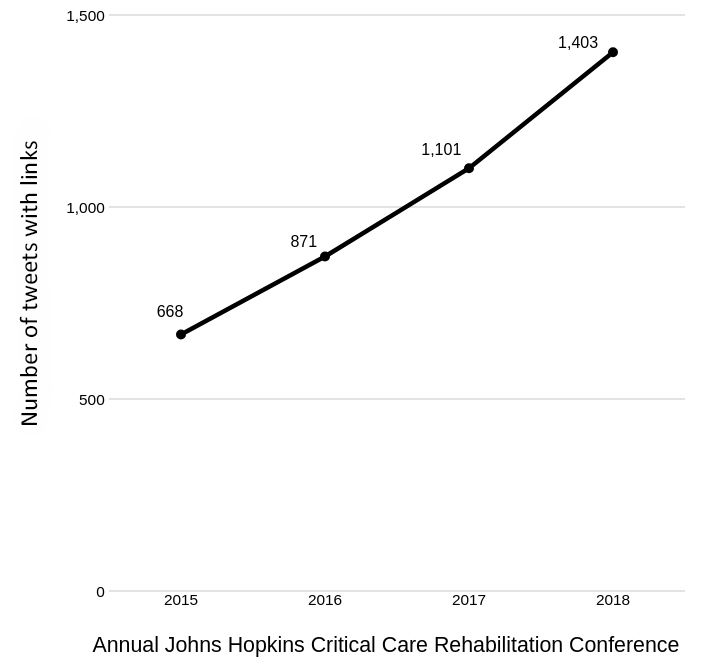
Number of tweets with links throughout history of annual Johns Hopkins Critical Care Rehabilitation Conference

**Figure 2:**
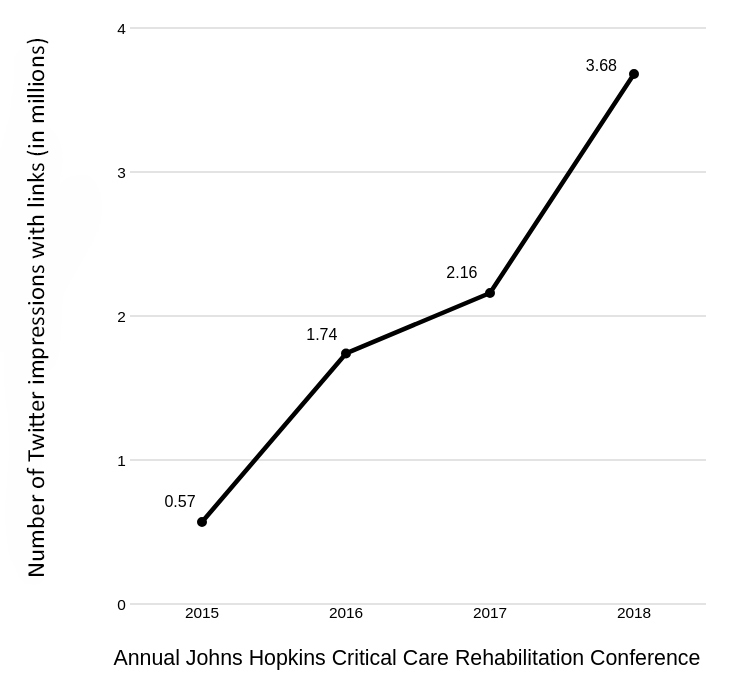
Number of impressions for tweets with links

In the days after the 2018 conference, reports were assembled by the social media chair using the Symplur Healthcare Hashtags platform [18] and Twitter’s metrics [19]. Symplur is a web platform and social project that identifies trends and presents data visualizations based on Twitter health care hashtags. This report demonstrated substantial growth in the number of tweets with hyperlinks to scholarly literature from the first conference in 2012 until the conference in 2018. This growth was associated with growth in the conference’s scope and size and addition of a librarian to the Social Media Ambassador Team starting in 2016. The conference content had more than quadrupled, from 29 presentations in 2012 to 123 presentations in 2018. There were 668 tweets in 2015 versus more than 1,400 tweets in 2018 that included hyperlinks to scholarly literature (Figure 1). Similarly, the number of impressions (i.e., a measure of the number of times a tweet is seen in a user’s feed [20]) for tweets with hyperlinks increased from approximately 570,000 in 2015 to more than 3.6 million in 2018 (Figure 2), implying that the tweets were reaching a larger audience. Because the librarian simplified and expedited the dissemination of references, the rest of the Social Media Ambassador Team was able to share additional content, thereby creating a collaborative and engaging environment focused on the conference’s content and events.

### Promoting information literacy: librarian as exhibitor

The content of health care continuing education conferences often reflects a discussion of methods and resources that fall within a librarian’s scope of knowledge. For instance, presenters may share reporting guidelines or methods for finding the best evidence. When presented with the opportunity to be part of the exhibitor hall, the librarian considered what information would be useful to this audience and created four handouts focused on: (1) working with evidence, (2) becoming an expert searcher, (3) critically appraising evidence, and (4) writing and publishing (Table 1). The handouts highlighted PubMed [21] along with other bibliographic literature databases, including CINAHL [22], Physiotherapy Evidence Database (PEDro) [23], and PsycINFO [24].

**Table 1:** Summary of available handouts

Handout title	Information contained
1. Work with Evidence	Reporting standards Databases and resources Evidence-based practice pyramid
2. Become an Expert Searcher	Tips and tricks for searching databases How to save searches Sample searches
3. Critically Appraise Research	Reasons why critical appraisal is important Critical appraisal checklists and tools
4. Write and Publish	Journal finder and suggester sites Tips for evaluating journals for credibility Impact metrics
5. Twitter for Academia	Benefits of using Twitter How to sign up for Twitter How to follow the conference tweets

Because attendees often were working on effecting change in their home institutions through structured quality improvement initiatives, the handouts also included a hyperlink to the Standards for Quality Improvement Reporting Excellence (SQUIRE) Guidelines [25, 26]. Each one-page handout contained hyperlinks to important resources, the librarian’s physical medicine and rehabilitation [27] and early mobility [28] LibGuides [29], as well as the librarian’s professional contact information for follow-up questions. In 2018, given the popularity of Twitter, the librarian added a fifth handout, “Twitter for Academia.” This newest handout outlined the benefits of using Twitter for academic and health care populations and provided instructions for signing up and following conference-related tweets.

Many conference attendees visited the librarian’s exhibitor table. Attendees were interested in the handouts, often taking multiple copies to distribute or post at their home institutions. As of 2018, some handouts had a Creative Commons license [30] to allow sharing and reuse with attribution.

**Figure 3:**
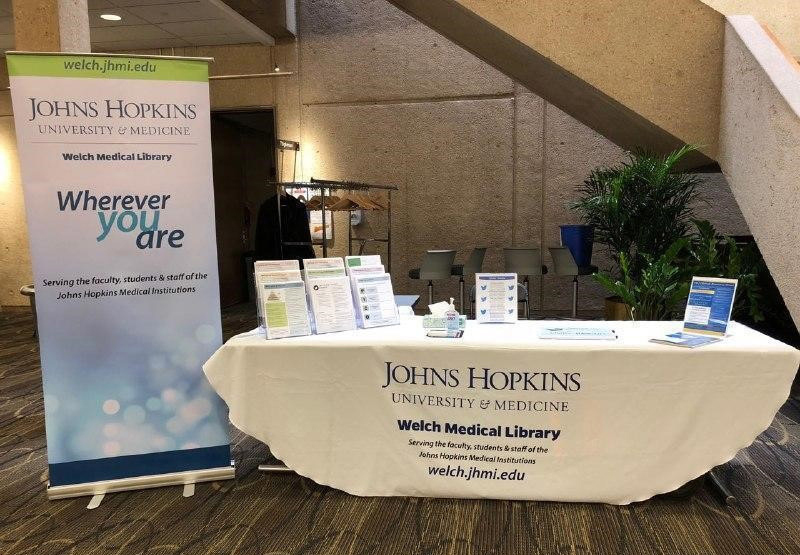
The librarian’s exhibitor hall table

The librarian used the Welch Medical Library’s branded tablecloth and banner and displayed the five handouts in clear resin holders for the exhibition table (Figure 3). There was also a display of the presenters’ Twitter handles. Some attendees and organizers took photos of the Twitter handles so that they could follow all Twitter-engaged presenters and tag them in their own tweets.

### Searching for evidence: librarian as presenter

In 2018, the conference organizers invited the librarian to be a presenter. The librarian was allotted fifteen minutes as part of a session on quality improvement, with a goal of promoting best practices for searching for evidence as well as sharing critical appraisal resources. A major component of the quality improvement process is translating existing evidence into practice [31], making skills in searching bibliographic literature databases and evidence appraisal paramount for effective quality improvement implementation.

For the presentation, the librarian devised a mnemonic to make the content more memorable (Table 2): “ABCDE” to outline the steps of an effective bibliographic literature database search approach. The ABCDE framework encouraged participants to Assess their topic, Brainstorm keywords, look for database Controlled vocabulary such as Medical Subject Headings (MeSH) and Emtree, Document the search, and critically appraise the Evidence. Additional context was provided for each step to fit within the quality-improvement process. The presentation concluded with screenshots of the librarian’s LibGuides, which have a curated selection of resources as well as bibliometric trend visualizations that the librarian and a colleague created [27–29].

**Table 2:** Searching for evidence for quality improvement projects mnemonic

Letter	Learning objective
A	Assess topic using patient/population, intervention, comparison/control, outcomes (PICO)
B	Brainstorm search terms
C	Use Controlled vocabulary
D	Document the search
E	Critically appraise Evidence

## DISCUSSION

Health sciences librarians have a long tradition of multidisciplinary collaboration. Many attend rounds or meetings with the goal of identifying scholarly literature to assist in health care decision-making; however, to our knowledge, there is not a published instance of a health sciences librarian being embedded in a health care conference with the roles of the librarian at the Annual Johns Hopkins Critical Care Rehabilitation Conference.

The conference organizers agreed that the librarian’s involvement was beneficial because it increased the reach of the conference and promoted research methods education. Although the librarian had witnessed the progression of early rehabilitation at an institutional level, it became obvious that there was global interest in early rehabilitation for critically ill patients. The librarian found the conference engaging and fulfilling in ways that differed from traditional librarianship, where typical user engagements happen in solitary environments at a desk, due to opportunities to engage in person.

### Lessons learned

Each year, the conference organizers and the librarian reflected on what changes should be made to enhance the work flow. Improvements since the librarian’s involvement began in 2016 have included communicating more clearly about which members of the Social Media Ambassador Team should cover which sessions and what content they should tweet. It also became important to set written guidance, including a suggested number of tweets per session per ambassador, with priority given to sharing the scholarly literature. A single ambassador was also designated to retweet #ICURehab content coming from external Twitter users so that the entire team did not have to become preoccupied with retweeting content. The librarian found that despite bringing hundreds of each handout, more were needed. It was critical that the number of handouts exceed the number of attendees due to some attendees collecting multiple copies to share. Finally, best practices for database searching and resources for critical appraisal were broad subjects to cover in only fifteen minutes in the 2018 conference; however, the primary goal was not to create a step-by-step guide, but rather to raise awareness of effective ways to search, appraise, and report.

### Future plans

While Twitter impact was measured through various reporting platforms, initial consideration was not given to measuring the librarian’s impact through exhibiting or presenting. Visitors to the table were not counted, and no attendees were surveyed on their opinions of having a librarian embedded in the conference. Future conferences could present opportunities to qualitatively or quantitatively measure the impact of these formal and informal methods of education to attendees.

In 2019, all of the librarian’s handouts had a Creative Commons license to encourage distribution and reuse. Because the conference grew rapidly and included multiple simultaneous sessions, a second librarian was added for the 2019 conference. The second librarian, a pediatric liaison, was familiar with literature in the pediatric track, allowing the first librarian to continue tweeting the adult track. The second librarian also joined the exhibitor table, creating more opportunities for networking and education.

## CONCLUSIONS

Librarians are an important part of the multidisciplinary team for evidence-based practice implementation and can add value when they are integrated into health care continuing education conferences. The Annual Johns Hopkins Critical Care Rehabilitation Conference is a case study of a health sciences librarian’s ability to contribute to advancing practice by efficiently and succinctly sharing scholarly literature through the Twitter platform, providing handouts on reporting guidelines and other resources, sharing methods for finding and publishing evidence, networking with attendees, and providing instruction on searching for evidence for quality improvement projects.

Other conferences could benefit from creating an embedded librarian role and collaborating with a librarian. Librarians’ expertise in information organization and dissemination make them valuable team members for health care conferences, helping to further evidence-based practice.

## COMPETING INTERESTS

Authors Sapna R. Kudchadkar, MD, PhD; Michael Friedman, PT, MBA; and Dale M. Needham, MD, PhD, are members of the Conference Planning Committee.

## 

**Carrie Price, MLS**, cprice17@jhmi.edu, https://orcid.org/0000-0003-4345-3547, Librarian, Welch Medical Library, Johns Hopkins University School of Medicine, Baltimore, MD

**Sapna R. Kudchadkar, MD, PhD**, sapna@jhmi.edu, Physician, Department of Anesthesiology and Critical Care Medicine, Department of Physical Medicine and Rehabilitation, and Department of Pediatrics, Johns Hopkins University School of Medicine, Baltimore, MD

**Pragyashree Sharma Basyal, BS**, prag.sharma@jhmi.edu, http://orcid.org/0000-0002-7180-6955, Staff, Division of Pulmonary and Critical Care Medicine, and Division of Geriatric Medicine and Gerontology, Johns Hopkins University School of Medicine, Baltimore, MD

**Archana Nelliot, MD**, anelliot@pennstatehealth.psu.edu, Resident, Department of Pediatrics, Penn State Hershey Medical Center, Hershey, PA

**Madison Smith, BS**, msmit354@jhmi.edu, Staff, Division of Pulmonary and Critical Care Medicine, Johns Hopkins University School of Medicine, Baltimore, MD

**Michael Friedman, PT, MBA**, mfried26@jhmi.edu, Physical Therapist, Department of Physical Medicine and Rehabilitation, Johns Hopkins Hospital, Baltimore, MD

**Dale M. Needham, MD, PhD**, dale.needham@jhmi.edu, Professor, Division of Pulmonary and Critical Care Medicine, Department of Physical Medicine and Rehabilitation, Johns Hopkins University School of Medicine, Baltimore, MD; and School of Nursing, Johns Hopkins University, Baltimore, MD
